# Bicaudal D1 impairs autophagosome maturation in chronic obstructive pulmonary disease

**DOI:** 10.1096/fba.2018-00055

**Published:** 2019-10-29

**Authors:** Nicolas Mercado, Thomas Colley, Jonathan R. Baker, Chaitanya Vuppussetty, Yuta Kono, Colin Clarke, Sharon Tooze, Terje Johansen, Peter J. Barnes

**Affiliations:** ^1^ Airway Disease Section National Heart and Lung Institute Imperial College London UK; ^2^ Novartis Institutes for BioMedical Research Basel Switzerland; ^3^ Pulmocide Ltd London UK; ^4^ London Research Institute Cancer Research UK London UK; ^5^ Molecular Cancer Research Group Institute of Medical Biology University of Tromsø – The Arctic University of Norway Tromsø Norway

**Keywords:** autophagosome maturation, cardiac glycosides, keap1, oxidative stress, p62

## Abstract

Bicaudal D1 (BICD1), an adaptor for the dynein‐dynactin motor complex, has been identified as a susceptibility gene in chronic obstructive pulmonary disease (COPD). Autophagy, an essential cellular homeostasis process, is defective in COPD, in which oxidative stress‐induced misfolded proteins accumulate into toxic aggregates dependent on the accumulation of the autophagic cargo receptor p62. Defective autophagy can be caused by mutations in the dynein and dynactin motor complex suggesting a possible link between BICD1 and defective autophagy in COPD. BICD1 levels were measured in peripheral lung tissue from COPD patients together with markers of autophagy and found to be increased in COPD together with autophagosomes, p62 and p62 oligomers. In vitro exposure of bronchial epithelial cells to cigarette smoke extracts (CSEs) revealed that high concentrations of CSE induced defective autophagosome maturation with accumulation of BICD1, p62 and ubiquitin‐associated p62 oligomers. This was confirmed in vivo using CS‐exposed mice. Furthermore, we identified that formation of CS‐induced p62 oligomers required an interaction with Keap1. Overexpression and ablation of BICD1 confirmed that increased BICD1 negatively regulates autophagosome maturation inducing accumulation of p62 and p62 oligomers and that it can be reversed by cardiac glycosides. We conclude that defective autophagosome maturation in COPD is caused by oxidative stress‐mediated BICD1 accumulation.

**Abbreviations**
AL: autolysosome
AP: autophagosome
BICD1: Bicaudal D1
COPD: chronic obstructive pulmonary disease
HBECs: human bronchial epithelial cells
HC‐CSE: high concentration cigarette smoke extract
LC3‐I: microtubule‐associated proteins 1A/1B light chain 3B
LC3‐II: phosphatidylethanolamine conjugated microtubule‐associated proteins 1A/1B light chain 3B

## 1 INTRODUCTION

Chronic obstructive pulmonary disease (COPD) is an age‐related condition caused by oxidative stress, usually resulting from long‐term cigarette smoking or biomass smoke exposure that, with no current drugs that can stop the progressive decline in lung function, is set to become the third leading cause of death worldwide.^1^

COPD has been associated with defective turnover of macromolecules damaged by oxidative stress that results in the accumulation of ubiquitinated protein aggregates (aggresomes). The association of aggresomes with the autophagic cargo receptor p62 suggests defective autophagy in COPD lung;^2^, ^3^, ^4^ however, other studies suggest that excessive activation of autophagy is involved in apoptosis and alveolar destruction in emphysema.^5^, ^6^, ^7^, ^8^, ^9^

Macroautophagy (hereafter referred to as autophagy) is an evolutionary conserved lysosomal degradation process for the elimination and recycling of damaged or surplus cytosolic components. This process involves the sequestration of cytoplasmic proteins and organelles into a double membrane vesicle called the autophagosome.^10^ During the maturation step, autophagosomes fuse with lysosomes to form autolysosomes, where the final hydrolytic degradation of the engulfed cytoplasmic material takes place.^10^ Distinct from the ubiquitin proteasome system (UPS), autophagy can selectively degrade large structures such as protein aggregates, intracellular bacteria, viral capsids, and protein aggregates.^10^, ^11^ A more unselective, bulk autophagy process is also induced by nutrient limitation. The formation of autophagosomes requires a well‐orchestrated activation of protein complexes that constitute the basal autophagy apparatus.^12^ Among these autophagy‐related (Atg) proteins are the Atg8 family proteins, including microtubule‐associated protein 1A/1B‐light chain 3 (LC3‐I) that, by conjugation to phosphatidylethanolamine, becomes LC3‐II and is integrated into autophagosomes.^13^ Selective autophagy receptors such as p62, NBR1, and NDP52 recognize ubiquitinated proteins and intracellular pathogens destined for autophagy and the cargo is directed toward developing autophagosomes where they bind LC3 via their LC3‐interacting regions.^14^ “Autophagic flux” is the term used to define the process of autophagosome synthesis (initiation), delivery of autophagic substrates to the lysosome and subsequent degradation of autophagic substrates inside the lysosome.^15^ Both the delivery of autophagosome to the lysosome and degradation are also called “autophagosome maturation.” The presence of autophagosomes can equally mean the activation of autophagy or defective autophagosome maturation. The mTOR inhibitor, rapamycin, is an inducer of autophagy and therefore a potential drug for the restoration of defective autophagy in various diseases.^16^ Whereas mTOR is involved primarily in the initiation steps of autophagy, the use of rapamycin for defects in autophagosome maturation could be potentially detrimental as shown in a murine model of amyotrophic lateral sclerosis where treatment with rapamycin induced further accumulation of protein aggregates and reduced cell viability.^17^, ^18^ Thus understanding the pathway involved in defective autophagic flux is crucial for developing therapies to reverse pathological conditions such as COPD.

Kong et al^19^ recently identified in a genome‐wide association study of emphysema an association of a single‐nucleotide polymorphism in bicaudal‐D1 (BICD1) with the presence or absence of emphysema. BICD1 is one of the two mammalian homologues of *Drosophila* bicaudal‐D1, acting as adaptor proteins that can regulate dynein‐based vesicle motility within the cytoplasm.^20^, ^21^ BICD1 is involved in microtubule anchorage at the centrosome and the regulation of microtubular function.^22^ Autophagosomes move in the direction of lysosomes located near the centrosome by association with the dynein motor complex.^23^ Kong et al^19^ showed that the locus most highly associated with emphysema is in a region that covers the second exon of the gene BICD1 that encodes the sequence located in the coiled‐coil domain at the N terminus of the protein, which directly interacts with dynein. The role of BICD1 in COPD and autophagy however remains unknown.

Our findings confirmed the presence of autophagosomes in lung tissue from COPD patients together with an accumulation of p62 and p62 oligomers and total LC3, suggesting a defect in autophagosome maturation. We also show that BICD1 is increased in COPD lung and accumulation of this protein results in defective autophagosome maturation. We recapitulated these findings using an in vitro cigarette smoke (CS) model within a bronchial epithelial cell line and found that, accumulation of p62, p62 oligomers and autophagosomes were observed at high concentrations of CS. In addition, p62 oligomers were found to be strongly associated with ubiquitinated substrates and that oligomerization depended on direct association with Keap1 during defective autophagosome maturation. Finally, cardiac glycosides, but not rapamycin, reversed the defective autophagosome maturation.

## 2 MATERIALS AND METHODS

### 2.1 Reagents and antibodies

Chloroquine, ammonium chloride, digoxin, digoxigenin, strophanthidin, DAPI mounting media, rapamycin, 3‐(4,5‐dimethylthiazol‐2yr)‐2‐5‐diphenyltetrazoliumbromide (MTT), actinomycin D, bafilomycin SMER28 and N‐acetylcysteine from Sigma (Poole, UK) were used.

Protein A magnetic beads were purchased from Thermo Scientific (East Riding of Yorkshire, UK). Antibodies against the following were used for immunoblotting: p62 (rabbit polyclonal; #P0067), LC3 (rabbit polyclonal; #L8918), Ubiquitin (rabbit polyclonal; #SAB1306222), NBR1 (rabbit polyclonal, #SAB2107031) from Sigma. p62 (mouse monoclonal; # sc‐28359), caspase‐3 (rabbit polyclonal; #sc‐136219) from Santa Cruz Biotechnology. Beta‐actin (mouse monoclonal; #ab8226), GFP (#ab1218), and LAMP1 (rabbit polyclonal; #ab24170) from Abcam. Cleaved caspase‐3 (rabbit polyclonal; #9661) from Cell Signalling (New England Biolabs). Keap1 (mouse monoclonal; #TA502059) and FLAG (mouse monoclonal; #TA50011‐100) from Origene).

### 2.2 Cell culture

BEAS‐2B cells (human bronchial epithelial) (ATCC,) were cultured as previously described.^24^ BEAS‐2B cells were serum starved 24 hours before stimulation. Normal human bronchial epithelial cells (HBEC) were purchased from Lonza (Basel, Switzerland) and cultured as specified by the company. Human epithelial kidney cell line HEK293 cells (ATCC) were grown in Dulbecco's modified Eagle's medium supplemented with 10% fetal calf serum supplemented with nonessential amino acids, 2 mmol/L L‐glutamine, penicillin (100 units/mL), and streptomycin (100 µg/mL). A549 cells (ATCC) were cultured as previously described.^25^ A549 cells were serum starved 24 hours before CSE treatment.

### 2.3 Peripheral lung tissue

Peripheral lung tissue from four nonsmokers (healthy volunteers) subjects with normal lung function, 10 smoker subjects with normal lung function, eight subjects with GOLD stage 1 (mild), eight with stage 2 (moderate), four with stage 3 (severe), and eight with stage 4 (very severe) COPD were obtained using a tissue bank linked to an established patient registry and kindly provided by Dr James Hogg and Dr Mark Elliot (University of British Columbia).^26^

### 2.4 Plasmids and transfections

BEAS‐2B cells were transfected with siRNA for nonspecific oligonucleotide control (NC) and Keap1 (Life Technologies) using HiPerfect (Qiagen) for 48 hours or 72 hours. HEK293 cells were transfected with different plasmids using Lipofectamine (Invitrogen). The following plasmids were characterized in a previous work by Lamark et al^27^: pDest‐EGFP‐p62 WT, pDest‐EGFP‐p62 ∆123‐170, pDest‐EGFP‐p62 ∆170‐256, pDest‐EGFP‐p62 ∆256‐370, pDest‐EGFP‐p62 ∆346‐385, pDest‐EGFP‐p62 (1‐385) that is ∆UBA (∆385‐440), pDest‐mCherry‐EGFP‐p62, pDest‐mCherry‐EGFP‐LC3B, and pDest‐EGFP‐p62 E352A (KIR point mutant). pBABE‐puro mCherry‐EGFP‐LC3B were a gift from Ana Maria Cuervo and pEGFP‐LC3B was purchased from Addgene.

### 2.5 Quantification of autophagososmes and autolysosomes

HEK293 was transiently transfected with pBABE‐puro mCherry‐EGFP‐LC3B (500ng) using 1 µL of lipofectamine in 100µl Opti‐MEM™ I Reduced Serum Medium (Invitrogen) for 48 hours. Cells were then incubated with LC‐CSE or HC‐CSE for 5, 24, and 24 hours plus another 24 hours in fresh media without CSE (48 hours). Autophagosomes (Yellow color) and Autolysosomes (Red color) were quantified using CellProfiler.

### 2.6 Preparation of cigarette smoke extracts

One full‐strength Marlboro cigarette with filter removed (Phillip Morris USA) was directed via a tube into 10 mL of serum‐free culture media using a peristaltic pump. Cigarette smoke extract (CSE) was then passed through a 0.2 µm filter to sterilize and remove particulate matter and was used immediately. The optical density was measured at 320λ (OD 0.85 ± 0.03). The concentration of CSE used was calculated as % v/v of CSE vs total volume of media.

### 2.7 Western blotting and immunoprecipitation

Protein extracts were prepared using RIPA buffer (Sigma: 150 mmol/L NaCl, 1.0% IGEPAL® CA‐630, 0.5% sodium deoxycholate, 0.1% SDS, and 50 mmol/L Tris, pH 8.0.) and completed with protease inhibitor (Roche, Welwyn Garden City, UK). N‐ethylmaleimide (NEM; 25 mmol/L, Sigma) was also added to the RIPA buffer for detecting ubiquitin linked to immunoprecipitation of p62. Immunoprecipitation was conducted with anti‐p62 in 300‐600 μg of whole‐cell lysate overnight at 4°C. Immunoprecipitates were captured with Protein A magnetic beads. After extensive washing, bound proteins were released by boiling in SDS‐PAGE sample buffer. Protein extracts (40 µg of immunoprecipitates) were analyzed by SDS‐PAGE (Invitrogen) and detected with Western Blot analysis by chemiluminescence (ECL Plus; GE Healthcare). Protein expression levels were expressed relative to β‐actin.

### 2.8 Immunocytochemistry

BEAS‐2B and HEK293 cells were grown on poly‐L‐lysine coated glass coverslips in 24‐well plates. After experimental procedures, the cells were washed with PBS and fixed in 4% paraformaldehyde in PBS for 15 minutes. Cells were then washed twice with PBS and permeabilized with ice‐cold methanol (−20°C, 10 minutes). BEAS‐2B cells were blocked in 5% (w/v) milk in PBS for 1 hour followed by 2 hours incubation with rabbit anti‐LC3 (1/50) and mouse LAMP1 (1/50) in 5% milk. After washing three times with PBS, cells were further incubated with Alexa fluor® 488 Donkey Anti‐Rabbit (1/400) and APC anti‐mouse antibodies (1/100) for 1 hour. Cells were then washed three times with PBS and mounted onto slides using Fluoroshield with DAPI. Confocal microscopy was performed using a Zeiss LSM‐510 microscope and the Zeiss LSM Image Browser software was used to generate images. In addition to single images, Z‐series stacks of 0.5 µm were taken in order to spatially resolve the position of autophagosomes within the cells using a 63X oil lens magnification. To visualize the XZ and YZ planes at a given point of the stack, orthogonal views were also taken. Quantification of total LC3 area per nuclei and the number of LC3 punctae per nuclei was assessed using Image J analysis.

### 2.9 Statistical analysis

Data are expressed as median ± SEM. Results were analyzed using *t* test and one‐ or two‐way ANOVA for repeated measures with Dunnett or Bonferroni posttests using the Graph Pad Prism Software (Prism). Clinical data were analyzed by Kruskal‐Wallis followed by Mann‐Whitney. *P* < .05 was considered statistically significant.

## 3 RESULTS

### 3.1 BICD1, LC3‐II, p62, and total LC3 are increased in COPD lungs

**Figure 1:**
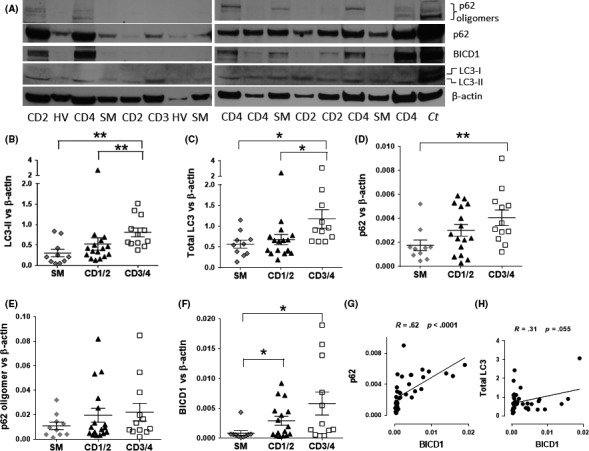
Increased p62, LC3‐II, total LC3, and BICD1 proteins in chronic obstructive pulmonary disease (COPD) patients. Lung tissue from surgical resections were obtained from four healthy volunteers (HV), 10 non‐COPD smoker volunteers (SM), 16 mild CD1/2 (COPD GOLD stage 1 plus GOLD stage 2), and 12 severe CD G3/4 (COPD GOLD stage 3 plus GOLD stage 4) and whole cell extracts were prepared for Western blot. (A) Representative blot. *Ct*: Control whole cell extract from BEAS‐2B cells. Immunoblotting for p62, LC3‐II, and BICD1 were performed and relative protein amounts were calculated and plotted on a graph against β‐actin: (B) LC3‐II, (C) LC3‐I + LC3‐II (total LC3) (D) p62, (E) p62 oligomers, and (F) BICD1. (G) Correlation between p62 and BICD1 in all patients. (H) Correlation between total LC3 and BICD1 in all patients. Data were analyzed by using Kruskal‐Wallis followed by Mann‐Whitney. *P* < .05 was considered statistically significant. *P < .05, **P < .01. Whole cell extracts from BEAS‐2B cells were used as control (*Ct*) in the Western blot membrane

Whole‐cell extracts were obtained from four healthy volunteers (HV), 10 healthy smokers (SM), 16 patients with mild COPD (CD1/2; GOLD1 plus GOLD 2), and 12 severe COPD (CD3/4; GOLD3 plus GOLD4) (Table 1). HV data were not used in the statistical analysis due to low numbers. The expression of markers of autophagy LC3‐II, total LC3 (I + II), and p62 was measured by Western blotting. As shown in previous studies,^3^, ^5^, ^6^ autophagosomes (LC3‐II) were significantly increased in CD3/4 patients compared to SM (*P* < .01), but also when compared to CD1/2 patients (*P* < .01) (Figure 1A,B). Although CD1/2 also displayed high levels of LC3‐II, these were not significantly different compared to SM (*P* = .12) (Figure 1A,B). LC3‐II accumulation measures the presence of autophagosomes whereas total LC3 (cytoplasmic LC3‐I and autophagosomal LC3‐II) and p62 can indirectly indicate a defect in autophagy maturation.^15^ Total LC3 protein levels in CD3/4 patients were significantly increased compared to SM (*P* < .05) and CD1/2 (*P* < .05) (Figure 1A,C). The accumulation of LC3‐II negatively correlated with % predicted FEV_1_ (forced expiratory volume in 1 second) in COPD patients (*r* = −0.56; *P* < .005) (Table 2). The expression of p62 was significantly increased in CD3/4 compared with SM (*P* < .01) and the increase in CD1/2 patients was also higher than smokers, although not significantly (*P* = .1) (Figure 1A,D). Interestingly, the expression of p62 negatively correlated with lung function by FEV_1_ (% predicted) in all COPD patients and only CD3/4 patients (Table 2). In addition, p62 oligomers were increased in CD1/2 and CD3/4 compared to SM; however, these were not significant (Figure 1A,E). A positive correlation between smoking history as measured by pack years versus p62 oligomers were observed in all smokers and COPD patients (*r* = 0.54; *P* < .01) (Table 2). CSEs have been shown to induce p62 and LC3 protein expression in bronchial epithelial cells^28^ so we compared the markers of autophagy between non/ex‐smokers versus current smokers (Figure S1). Our data suggest that current smokers do not display higher levels of LC3 and p62 expression as compared to nonsmokers.

**Table 1:** Patient's profile

	HV	SM	COPD gold1/2	COPD gold3/4
Age	55.6 ± 13.2	65.8 ± 4.0	65.2 ± 2.1	60.3 ± 1.7
Sex (M/F)	2/2	4/6	9/6	5/7
Smoking (pack‐year)	0	58 ± 10	56 ± 9 (n = 15)	46 ± 6 (n = 10)
pre‐FEV1 (L)	2.83 ± 0.40	2.42 ± 0.19	2.06 ± 0.16	0.79 ± 0.17 (n = 11)***
post‐FEV1 (L)	2.98 ± 0.49	2.57 ± 0.19	2.18 ± 0.16	0.86 ± 0.16***
pre‐FVC (L)	3.60 ± 0.60	3.36 ± 0.28	3.40 ± 0.25	1.99 ± 0.28 (n = 11)*
post‐FVC (L)	3.71 ± 0.68	3.49 ± 0.27	3.56 ± 0.27	2.24 ± 0.28 (n = 11)*
FEV1 (%pred)	98 ± 7	96 ± 5	76 ± 4	28 ± 5****
FEV1/FVC (%)	81 ± 2	74 ± 1	62 ± 2*	62 ± 3 (n = 11)****
PO2 (mmHg)	116 (n = 2)	107 ± 22 (n = 4)	105 ± 11 (n = 13)	63 ± 4 (n = 9)* ^,^ a
O2SAT	0.98 (n = 2)	0.95 ± 0.02 (n = 4)	0.97 ± 0.01 (n = 13)	0.92 ± 0.03 (n = 5)
PCO2 (mmHg)	40.5 (n = 2)	43 ± 1 (n = 4)	48 ± 2 (n = 13)	53 ± 2 (n = 8)
HCO3 (mmol/L)	25 (=2)	23 ± 1 (n = 4)	25 ± 0.4 (n = 13)	28 ± 3 (n = 4)
PH	7.41 (n = 2)	7.3 ± 0.1 (n = 4)	7.3 ± 0.02 (n = 13)	7.4 ± 0.02 (n = 8)

Abbreviations: HV, healthy volunteers; SM, Non‐COPD smokers.

a Significant in COPD3/4 vs COPD1/2. Nonparametric Kruskal‐Wallis followed by Dunn's multiple comparison tests (only showing SM vs COPD1/2 or COPD3/4).

**** *P* < .0001.

*** *P* < .001.

* *P* < .05.

**Table 2:** Correlations

	SM			COPD Gold 1/2			COPD Gold 3/4			All COPD		
	FEV_1_	FEV_1_/FVC	SM Pack‐y	FEV_1_	FEV_1_/FVC	SM Pack‐y	FEV_1_	FEV_1_/FVC	SM Pack‐y	FEV_1_	FEV_1_/FVC	SM Pack‐y
p62	*R* = −.22 *P* = .54	*R* = −.19 *P* = .61	*R* = .36 *P* = 31	*R* = −.42 *P* = .10	*R* = .30 *P* = .25	*R* = .07 *P* = .81	***R* = −.60** ***P* = .043** *	*R* = −.31 *P* = .33	*R* = .06 *P* = .88	***R* = −.45** ***P* = .017** *	*R* = −.11 *P* = .59	*R* = −.04 *P* = .85
p62 dimer	*R* = .19 *P* = .61	*R* = .36 *P* = .32	*R* = .49 *P* = .15	*R* = −.36 *P* = .17	*R* = −.29 *P* = .27	***R* = .82** ***P* = .0004** ***	*R* = −.52 *P* = .094	*R* = −.30 *P* = .34	*R* = −.16 *P* = .66	*R* = −.27 *P* = .16	*R* = −.21 *P* = .24	***R* = .54** ***P* = .006** **
LC3‐II	*R* = −.37 *P* = .30	*R* = −.2 *P* = .57	*R* = .07 *P* = .87	*R* = −.35 *P* = .18	*R* = .32 *P* = .22	*R* = .18 *P* = .53	*R* = .09 *P* = .78	*R* = .01 *P* = .99	*R* = .18 *P* = .62	***R* = −.56** ***P* = .002** **	*R* = −.36 *P* = .056	*R* = −.12 *P* = .57
Total LC3	*R* = −.33 *P* = .34	*R* = −.26 *P* = .47	*R* = −.13 *P* = .73	*R* = −.35 *P* = .18	*R* = .06 *P* = .82	*R* = −.24 *P* = .39	*R* = .07 *P* = .83	*R* = .0 *P* = 1.0	*R* = .04 *P* = .93	***R* = −.38** ***P* = .045** *	***R* = −.38** ***P* = .044** *	*R* = −.15 *P* = .47
BICD1	*R* = −.14 *P* = .7	*R* = .11 *P* = .76	*R* = .20 *P* = .58	*R* = −.48 *P* = .06	*R* = .18 *P* = .51	*R* = .37 *P* = .17	***R* = .59** ***P* = .049** *	*R* = .43 *P* = .17	*R* = .01 *P* = .99	***R* = −.45** ***P* = .017** *	*R* = −.20 *P* = .31	*R* = .15 *P* = .46

Abbreviations: HV, healthy volunteers; SM, Non‐COPD smokers.

*Note*: Spearman correlation (nonparametric).

*** *P* < .001.

** *P* < .01.

* *P* < .05. Bold values simply highlights correlations that are statistically significant.

We observed a significant increase in BICD1 in CD1/2 and CD3/4 as compared to SM (Figure 1A,F). BICD1 positively correlated with p62 monomers (*r* = 0.76, *P* < .0001) (Figure 1G) and was close to significance with total LC3 (*r* = 0.31, *P* < .055) (Figure 1H). In addition, BICD1 negatively correlated with lung function by FEV_1_ (% predicted) in all COPD patients and only CD3/4 patients (Table 2). Together these data suggest that although autophagosomes are increased as COPD severity progresses, this might be due to a defect in autophagosome maturation resulting in increased total LC3 and p62 together with evidence of p62 oligomer formation. Our data also suggest that an increase in BICD1 in milder CD1/2 precedes the accumulation of p62 and total LC3 observed in more severe CD3/4.

### 3.2 Cigarette smoke impairs autophagic clearance of p62 oligomers and autophagosomes

**Figure 2:**
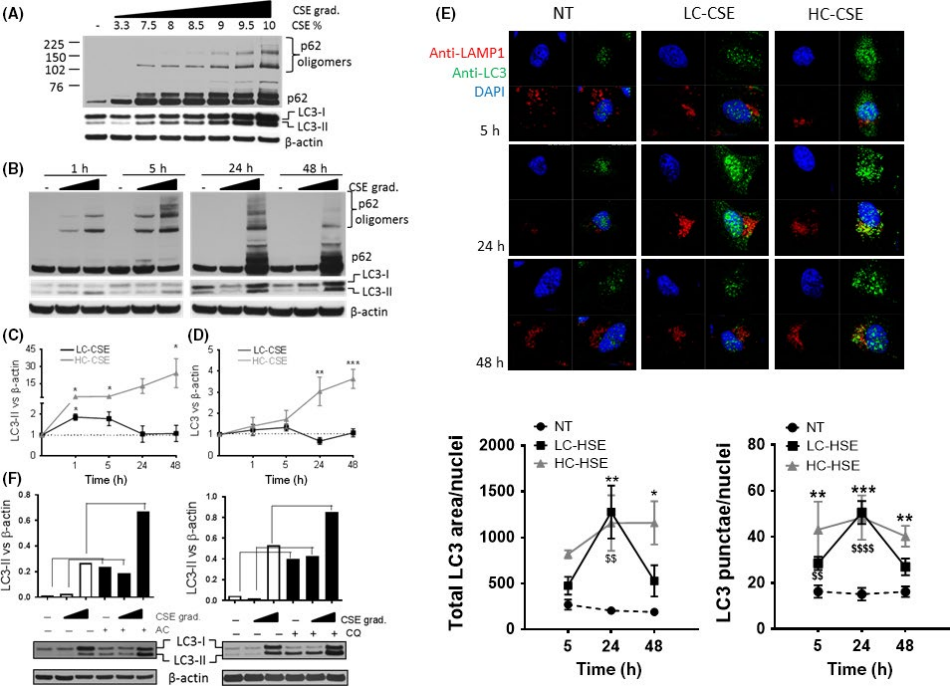
Increased p62, LC3‐II, and total LC3 in bronchial epithelial cell line (BEAS‐2B) treated with cigarette smoke extract (CSE). (A) Whole‐cell extracts from BEAS‐2B cells that were treated with increasing concentration of CSE (3.3%‐10% v/v) for 24 h were analyzed by immunoblotting for LC3, p62, and β‐actin, n = 3 (B) Whole‐cell extracts from BEAS‐2B cells treated with a low and high concentration of CSE (LC‐CSE (3.3% v/v) and HC‐CSE (10% v/v)) at increasing time points (1‐48 h) and analyzed by immunoblotting for LC3, p62, and β‐actin, n = 4. (C) Autophagosomes (AP) formation was assessed by LC3‐II vs β‐actin from (B) and plotted. (D) Total LC3 proteins levels were assessed against β‐actin from (B). For (C) and (D) statistical analysis was performed using two‐way ANOVA for repeated measures with Bonferroni posttests. * *P* < .05, ** *P* < .01, *** *P* < .001. (E) APs were detected by confocal microscopy using a rabbit anti‐LC3 antibody conjugated to 488 Donkey Anti‐rabbit and lysosomes were detected by using mouse anti‐LAMP1 antibody conjugated to anti‐mouse APC. DAPI was used to visualize nuclei. Cells were incubated with CSE as described in (B) Scale bar: 10 μm. Statistical analysis was performed by comparing HC (*) and LC‐CSE ($) vs NT control *P* < .05, *P* < .01, *P* < .0001. Data are expressed as median ± SD of n = 3. * means comparing NT vs HC‐CSE; * *P* < .05, ** *P* < .01, *** *P* < .001. (F) An “LC3 turnover” assay was performed by incubating BEAS‐2B cells with LC‐CSE and HC‐CSE for 19 hours plus 5 hours in the presence of ammonium chloride (AC, left graph, 10mM) or chloroquine (CQ, right graph, 100 µmol/L). The amount of LC3‐II was plotted against β‐actin. Data are expressed as median ± SEM

In order to understand how cigarette smoke exposure affects the autophagic process in the lung, bronchial epithelial cells (BEAS‐2B cells) were incubated with increasing concentrations of CSE and markers of autophagy (LC3‐II, total LC3, and p62) were assessed by Western blotting. CSE induced a concentration‐dependent increase in autophagosomes (LC3‐II) 24 hours postexposure (visible from CSE 7.5%) (Figure 2A). As previously observed in COPD patients, CSE also induced a concentration‐dependent increase in p62 oligomers which were resistant to the reducing conditions of the SDS‐PAGE (Figure 2A). This increase in p62 oligomers correlated with an increase in total LC3. At higher concentrations of CSE (HC‐CSE [9%‐10%]), the increase in total LC3 and p62 oligomers are particularly visible (Figure 2A), suggesting a defect in autophagy maturation at high concentration of CSE only. CSE induced a concentration‐dependent decrease in cell viability with a maximum of 34% at HC‐CSE (*P* < .001) (Figure S2). An increase in cleaved caspase‐3 at HC‐CSE but not with lower CSE concentration suggests that apoptosis could be responsible for the reduction in cell viability observed (Figure S3). In order to compare a CSE concentration that induces autophagy versus a concentration that seemed to induce defective autophagic flux, BEAS‐2B cells were exposed to low concentration of CSE (LC‐CSE or 3.3%) and high concentrations (HC‐CSE or 10%) for 1, 5, 24 hours, and an additional time point 24 hours after washing cells with PBS and incubating with fresh media (48 hours). Both LC‐ and HC‐CSE induced autophagy (1‐5 hours) as shown by the conversion of LC3‐I to LC3‐II, suggesting an increase in LC3‐II incorporation into autophagosomes (Figure 2B,C). Whereas LC‐CSE induction of LC3‐II goes back to baseline levels at 24 hours, the increase in autophagosomes by HC‐CSE is further increased with time, despite the CSE stimulus having been removed (48 hours) (Figure 2B,C). These findings were confirmed using confocal microscopy by detection of autophagosomes using a specific antibody against LC3 which was conjugated to GPF (Figure 2E). Anti‐LAMP1 was used to localize lysosomes (conjugated to Alexa Fluor 594) (Figure 2E). LC‐CSE induced autophagosomes (LC3 punctae and total LC3 area), peaking at 24 hours and returning to baseline levels after cells were washed and incubated in fresh media after 24 hours (48 hours time point) (Figure 2E). HC‐CSE also induced autophagosomes; however, these failed to return to basal levels as shown at the 48 hours time point as compared to NT (total LC3 area and LC3 punctae) (Figure 2E). Hence, HC‐CSE induced persistent autophagosomes that were not cleared even 48 hours posttreatment, suggesting a lack of autophagosome clearance as a result of defective autophagosome maturation. In addition to p62 monomer accumulation, CSE also induced a concentration‐dependent increase in p62 oligomers that were resistant to the reducing conditions of SDS‐PAGE (Figure 2B). Similar to LC3‐II, LC‐CSE induced p62 oligomers that peaked at 5 hours and returned to basal levels at 24 hours. At HC‐CSE, p62 oligomers peaked between 5 hours and 24 hours but remained elevated at 48 hours (Figure 2B). p62 monomers were also increased at 24 hours and 48 hours time points. Preincubation with the antioxidant N‐acetyl cysteine (NAC, 10 mmol/L) totally prevented CSE‐induced p62, p62 oligomers, LC3‐II, and accumulation of total LC3, thus demonstrating that oxidants present in CSE are responsible for autophagy induction and accumulation of autophagic adaptors (Figure S4).

### 3.3 CSE impairs the autophagic flux by inhibiting autophagy maturation

**Figure 3:**
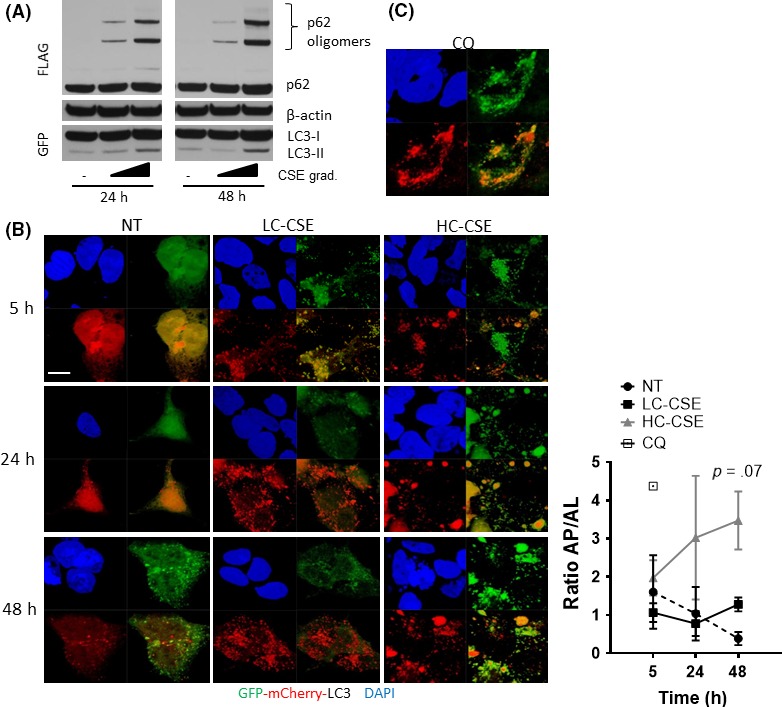
Evidence of defective autophagosome maturation in CSE‐treated HEK293 cells. (A) HEK293 cells were transfected with p62‐FLAG and LC3‐GFP and incubated with low or high CSE (LC‐CSE and HC‐CSE) for 24 h. Cells were washed and incubated with fresh media without CSE for another 24 h (48 h). Whole‐cell extracts were resolved by Western blot and immune‐stained against GFP and FLAG. (B) HEK293 cells were transfected with GFP‐mCherry‐LC3 plasmid and incubated with LC‐CSE or HC‐CSE for 5 h, 24 h, and 24 h plus another 24 h in fresh media without CSE (48 h). GFP and mCherry were visualized by confocal microscopy. DAPI was used as a nuclear control. Scale bar: 10 μm. The ratio of autophagosomes vs autolysosomes (AL/AP) was calculated and plotted. HC‐CSE vs NT at 48 h; *P* = .07 (C) HEK293 cells were transfected with GFP‐mCherry‐LC3 plasmid incubated with CQ for 5 h. Data are representative of three independent experiments

LC3‐II is normally present at either side of the double membrane of autophagosomes. LC3‐II present inside the autophagosome is digested with the internal cargo whereas LC3‐II present outside is recycled back into LC3‐I in the cytoplasm. If the autophagic flux is impaired at the maturation step, total levels of LC3 (LC3‐I + LC3‐II) are increased due to newly synthesized LC3 and a lack of LC3‐II degradation.^29^ After exposure to HC‐CSE, total LC3 was significantly increased at 24 hours and 48 hours compared to LC‐CSE and control suggesting defective autophagy maturation (Figure 2B,D). An “LC3 turnover” assay was performed in order to assess the autophagic flux at the initiation step. In the presence of the lysosomotropic compounds ammonium chloride (AC) or chloroquine (CQ) for 5 hours, baseline autophagic flux can be observed by an accumulation of autophagosomes (LC3‐II) (Figure 2F). Accumulation of autophagosomes still happens when cells were treated with LC‐CSE for 24 hours plus 5 hours with lysosomotropic compounds (Figure 2F). Interestingly, despite high levels of HC‐CSE‐induced LC3‐II, autophagosomes are still being formed suggesting that the early steps of the autophagic flux (initiation) are fully functional (Figure 2F). These findings were confirmed when using the inhibitor of lysosome to autophagosome fusion, bafilomycin, for 2 hours after HC‐CSE treatment (Figure S5). Our data imply that cells treated with HC‐CSE could be defective in autophagosome maturation such as the binding of autophagosomes to lysosomes to form autolysosomes. In order to detect if autolysosmes were visible in CSE‐incubated cells, BEAS‐2B cells were co‐stained with LAMP1 (lysosomal marker) and LC3 (Figure 2E); however, no co‐localization was observed. We also transiently transfected HEK293 cells with p62‐FLAG and GFP‐LC3 plasmids in order to optimize CSE concentrations that recreated normal CSE‐induced autophagy activation and CSE‐induced defective autophagic flux (Figure 3A). LC‐CSE induced LC3‐II and p62 oligomer accumulation 24 hours posttreatment, and, as seen in BEAS‐2B, these were partially reduced 24 hours after the CSE stimulus was removed (48 hours). In contrast, HC‐CSE strongly induced p62 oligomer accumulation at 24 hours, and this increase continued even after the CSE stimulus was removed (48 hours) (Figure 3A). p62 monomers were also increased. As expected, autophagosome (LC3‐II) formation followed a similar trend, with an accumulation at 24 hours that was further increased after 24 hours in fresh media (Figure 3A). This was associated with an overall increase in total LC3 protein (Figure 3A). Thus, HC‐CSE induced defective autophagy maturation in transfected HEK293 cells. In order to confirm if defective autophagy maturation could be visualized by microscopy, HEK293 cells were transiently transfected using a GFP‐mCherry‐LC3 tandem construct. Using this construct, autophagosomes and autolysosomes were labeled with yellow (mCherry and GFP) and red (mCherry only) signals, respectively. If autophagy is activated, both yellow and red punctae are increased. However, if autophagosome maturation into autolysosomes is blocked, only yellow punctae are increased without a concomitant increase in red punctae. We observed that at LC‐CSE autophagosomes were visible (yellow punctae) after 5 hours incubation and that they progressively became autolysosomes (red punctae) over time (24‐48 hours) (Figure 3B). HC‐CSE induced greater autophagosomes formation (yellow punctae, 5 hours) that remained constant over time with little evidence of autophagosome conversion to autolysosomes (red punctae) (Figure 3B). Quantification of autophagosomes (AP, yellow punctae) and autolysosomes (AL, red punctae only) suggest a persistence of autophagosomes over autolysosomes (Figure 3B; graph) as compared to control and similar to CQ after 5 hours. HC‐CSE induced some large structures that could not be autophagosomes. These likely represent p62 aggregates that have recruited GFP‐mCherry‐LC3. It has previously been shown that p62 can recruit GFP‐LC3 into protein aggregates in an autophagy‐independent manner.^30^ Interestingly, when HEK293 cells were incubated with chloroquine, to inhibit the binding of autophagosome to lysosome, the continuous presence of autophagosomes was observed, similar to the treatment with HC‐CSE (yellow punctae) (Figure 3C). Hence, a lack of autolysosome formation suggests that autophagosomes are unable to bind lysosomes after incubation with high concentration of CSE resulting in malfunctioning autophagic flux by defective autophagosome maturation.

### 3.4 Impairment of autophagic clearance of LC3 and p62 oligomers by high concentration of CSE confirmed in primary human bronchial epithelial cells

**Figure 4:**
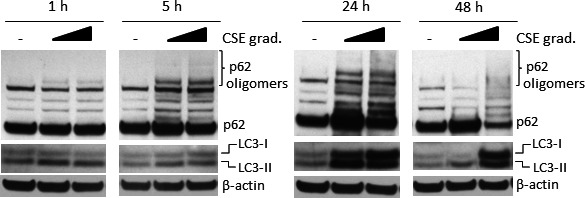
Defective autophagosome maturation in CSE‐treated primary human bronchial epithelial cells. HBECs from a healthy volunteer were treated with low or high CSE (LC‐CSE (20% v/v) and HC‐CSE (30% v/v)) at increasing times (1‐48 h) and analyzed by immunoblotting for LC3, p62, and β‐actin. At the 48 h time point, cells were washed and incubated with fresh media without CSE

We confirmed these observations in primary HBECs. HBECs were incubated with LC‐CSE and HC‐CSE for a period of 1, 5, and 24 hours and a further 24 hours in minimal media without CSE (48 hours). At both concentrations of CSE, early LC3‐II was observed (1‐5 hours) with marked increase in p62 oligomers after 5 hours exposure (Figure 4). After 24 hours incubation both LC‐CSE and HC‐CSE induced a strong accumulation of LC3‐II, total LC3, and p62 oligomers (Figure 4). However, at the 48 hours time point, basal levels of LC3 and p62 oligomers were observed with LC‐CSE suggesting a resolution of autophagy and subsequent clearance of autophagososmes. On the contrary, cells incubated with HC‐CSE showed a continuous presence of p62 oligomers and LC3‐II and increased total LC3 at 48 hours, suggesting impaired autophagic flux (Figure 4).

### 3.5 CSE‐induced p62 oligomers depend on the Keap1 interacting region

**Figure 5:**
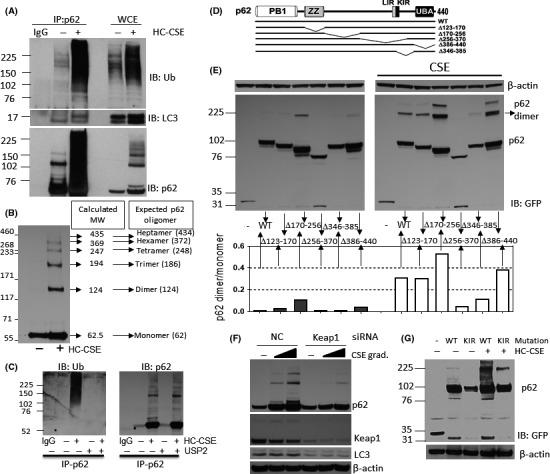
CSE induces p62 oligomers via their Keap1 interaction region (KIR). (A) BEAS‐2B cells were incubated with HC‐CSE for 24 hours and p62 was immunoprecipitated (IP) from whole‐cell extracts. IP‐p62 species were resolved by SDS‐PAGE followed by Western blot (WB) and membranes immune‐stained against ubiquitin (Ub), LC3, and p62. IgG control (no whole‐cell lysates used). (B) Molecular weight characterization of p62 oligomers. HEK293 cells were transfected with p62‐FLAG and incubated with HC‐CSE for 24 h. Whole‐cell extracts were resolved by WB and immune‐stained against FLAG. A molecular marker for high molecular weights (MW) was used. A graph extrapolating distance of bands from origin and MW determined the expected size of p62 oligomers and the possible combinations. (C) Deubiquitination assay of IP‐p62. BEAS‐2B cells were prepared as indicated in (A). Samples were divided by two and p62 was IP. IP‐p62 beads were incubated in deubiquitin buffer plus/minus Ubiquitin specific peptidase 2 (USP2). (D) Schematic representation of p62 functional domains: PB1, ZZ (ZZ‐type zinc finger domain), LIR, KIR, and UBA and schematic representation of the different deletion p62 mutants used in (E). (E) HEK293 cells were transfected with either WT p62‐GFP, or the following p62‐GFP deletion plasmids: ∆123‐170, ∆170‐256, ∆256‐370, ∆346‐385, and ∆UBA (∆386‐440) and incubated with HC‐CSE for another 24 h. WB was performed and membranes were immune‐stained using anti‐GFP and β‐actin. (F) BEAS‐2B cells were transfected with siRNA for Keap1 or a random oligonucleotide control (NC) and incubated with LC‐ and HC‐CSE for a further 24 h. WB was performed and membranes were immune‐stained using against p62, LC3, Keap1, and β‐actin. (G) HEK293 cells were transfected with either WT p62‐GFP or a KIR deletion p62‐GFP plasmid and incubated with HC‐CSE for another 24 h. WB was performed and membranes were immune‐stained using anti‐GFP and β‐actin. Data are representative of at least two independent experiments

p62 acts as a cargo receptor for the autophagic degradation of ubiquitinated substrates. p62 contains an N‐terminal PB1 domain that allows polymerization, a LIR motif interacting with LC3, a C‐terminal UBA domain that interacts with ubiquitin and a Keap1 interacting region (KIR) that interacts with Keap1 thus controlling Keap1 stability (Figure 5D).^31^, ^32^ CSE‐induced p62 oligomers, that are resistant to SDS‐PAGE reducing conditions, have been observed in macrophages from smokers and after hydrogen peroxide treatment.^33^, ^34^ The characteristics of these oligomers, their role in the flux of autophagy and the amino acid sequence of p62 that is responsible for the oligomer formation remain unknown. Endogenous p62, immunoprecipitated from BEAS‐2B cells that were incubated with HC‐CSE, displayed association with polyubiquitinated substrates (Figure 5A). The UBA region of p62 is known to bind polyubiquitin and is needed to form cytoplasmic bodies.^35^ p62 was also associated with LC3‐II at baseline levels, possibly as part of the basal autophagic flux. After HC‐CSE incubation, binding did not increase despite the presence of more LC3‐II (Figure 5A). Interestingly HC‐CSE also induced binding to cytoplasmic LC3‐I (Figure 5A). This is consistent with the recruitment of GFP‐mCherry‐LC3 to p62 aggregates seen in Figure 3B. NBR1 is another autophagy adaptor that contains a PB1 domain allowing heteropolymerization with p62. We found that p62 is associated with NBR1 but that the association was not increased after HC‐CSE incubation. In fact, NBR1 neither form SDS‐PAGE‐resistant oligomers, nor did it accumulate like p62 (Figure S6). Using 3%‐8% Tris‐acetate gels and a higher molecular weight ladder, we separated FLAG‐p62 that were overexpressed in HEK293 cells in order to predict the size of the oligomers induced by CSE. Despite FLAG's molecular size of 13kDa, p62 monomer was found at 62.5kDa mark (Figure 5B). In total six p62 oligomers were identified as possible dimers, trimers, tetramers, hexamers, and heptamers (Figure 5B). Incubation of IP‐p62 from CSE‐treated BEAS‐2B cells with the deubiquitinase USP2 also showed that deubiquitination did not result in reduced CSE‐induced p62 oligomers but reduced p62 association with ubiquitinated substrates (Figure 5C). In order to map the amino acid region of p62 involved in oligomer formation we used different GFP‐p62 deletion plasmids that were expressed in HEK293 followed by incubation with CSE (Figure 5E). As p62 expression changed according to the functional domain being deleted, we assessed p62 oligomer formation as p62 dimer versus monomer (Figure 5E). We found that deletion of amino acids 170 to 256 (**Δ**170‐256) actually increased dimer formation at baseline (about eightfold) and that deletion of the UBA (**Δ**386‐440) domain induced p62 dimer formation by about threefold. CSE induced p62 dimers by about 30‐fold in WT GFP‐p62. CSE also further induced GFP‐p62 (**Δ**170‐256) dimer formation compared to WT (by 60%). Interestingly, GFP‐p62 deletion plasmids **Δ**346‐385 and particularly **Δ**256‐370 prevented CSE‐induced dimer formation suggesting that one or more amino acids contained in these sequences are important for oligomer formation. Both GFP‐p62 **Δ**346‐385 and **Δ**256‐370 have in common the amino acid deletion sequence between 346 and 370 that includes the KIR (344‐356). This region is important for p62 direct association with Keap1 and subsequent autophagy‐mediated degradation. It is possible that amino acids contained in the KIR are responsible for CSE‐induced p62 oligomers. A549 lung cancer cell line, which harbors mutated Keap1 (G333C), is unable to bind to p62.^36^ We found that CSE only mildly induced p62 dimer formation in A549 cells, but did not induce any oligomers despite increasing LC3‐II and also LC3‐I at the highest concentrations (Figure S7). The importance of p62 interaction with Keap1 was confirmed by knocking down Keap1 in BEAS‐2B cells and looking at the effect on CSE‐induced p62 oligomers. Ablation of Keap1 reduced CSE‐induced oligomers and monomers (Figure 5F). Confirmation was obtained by looking at the effect of HC‐CSE on GFP‐p62 KIR mutant in HEK293 cells. CSE‐induced oligomers were dramatically decreased in HEK293 expressing the p62 KIR mutant (Figure 5G).

### 3.6 BICD1 expression is involved in defective autophagosome maturation

**Figure 6:**
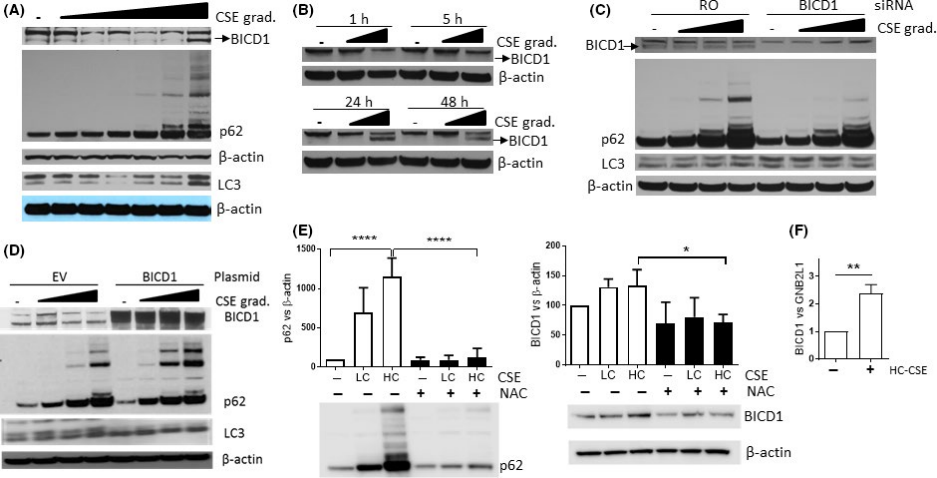
CSE induces BICD1 thus inhibiting autophagosome maturation. (A) Whole‐cell extracts from BEAS‐2B cells were treated with increasing concentrations of CSE (1.5%‐10% v/v) for 24 h and analyzed by immunoblotting for LC3, p62, BICD1, and β‐actin. The BICD1, p62, and β‐actin pictures were obtained from a 3%‐8% Tris‐acetate gel. The LC3 and (lower) β‐actin pictures were obtained from the same samples but using a 4%‐12% Bis‐tris gel. (B) Whole‐cell extracts from BEAS‐2B cells treated with LC‐ and HC‐CSE at increasing times (1‐48 h) and analyzed by immunoblotting for BICD1 and β‐actin. (C) BEAS‐2B cells were transfected with siRNA for BICD1 for 48 h or a random oligonucleotide control (NC) and incubated with increasing concentrations of CSE for a further 24 h. Western blot was performed and membranes were immune‐stained against p62, LC3, BICD1, and β‐actin. (D) BEAS‐2B cells were transfected either with an empty mCherry vector (EV) or with a mCherry‐BICD1 plasmid prior to incubation with increasing concentrations of CSE for a further 24 h. Western blot was performed and membranes were immune‐stained against LC3, BICD1, p62, and β‐actin. Data are representative of at least two independent experiments. (E) BEAS‐2B cells were preincubated with NAC (10 mmol/L) for 30 min prior to stimulation with LC‐ and HC‐CSE for 24 h. Western blot was performed and membranes were immune‐stained against BICD1, p62, and β‐actin. N = 3, *P < .05, ****P < .0001 using ANOVA with multiple comparisons. (F) BEAS‐2B cells were treated with HC‐CSE (10%) for 24 h. mRNA expression of BICD1 was determined and normalized to GNB2L1 mRNA levels. **P < .01

Having identified an increased BICD1 expression in COPD biopsies which was associated with p62 expression (Figure 1), we investigated the role of BICD1 as a possible protein involved in defective autophagy maturation. We looked at the concentration‐dependent effect of CSE on BICD1. Although two bands were visible, an increase in the lower band only happened when concentrations of CSE reached 10% (Figure 6A). This correlated with evidence of impaired autophagic flux (increased LC3 and p62 oligomers) (Figure 6A). In addition, HC‐CSE‐induced BICD1 was observed only at 24 and 48 hours time points, when autophagosome maturation was defective (Figures 6B and 2B). Defective autophagic flux could be responsible for BICD1 accumulation, hence when BICD1 was knocked down (KD) and cells incubated with increasing concentrations of CSE (3.3%‐10%), the accumulation of p62 oligomers was actually decreased as compared to random oligonucleotide control (RO) thus suggesting that BICD1 is a negative regulator of autophagy (Figure 6C). siRNA treatment increased LC3‐II levels so we used p62 to assess autophagy (Figure 6C). Interestingly, BICD1 knockdown only reduced the lower of the two bands observed thus identifying the lower band as BICD1 protein (Figure 6C). Overexpression of BICD1, as compared to an empty vector (EV), confirmed that BICD1 plays a role in impairing autophagic flux as CSE‐induced p62 oligomers were visibly increased (Figure 6D). LC3‐II levels were clearly increased with overexpressed BICD1 as compared to EV possibly suggesting a complete impairment of the autophagic flux (Figure 6D). Preincubation with the antioxidant NAC totally prevented CSE‐induced p62, p62 oligomers, LC3‐II, and accumulation as shown in Figure S4. We also established that NAC prevented an increase in BICD1 protein levels (Figure 6E). In fact, BICD1 expression was induced by CSE at the gene expression level (Figure 6F). Furthermore, pretreatment with actinomycin D reduced HC‐CSE‐induced BICD1 protein expression and prevented LC3‐II as well as reduced p62 monomers and oligomers thus averting defective autophagosome maturation (Figure S8).

### 3.7 p62, LC3, and BICD1 are increased in the lungs of CS‐exposed mice

**Figure 7:**
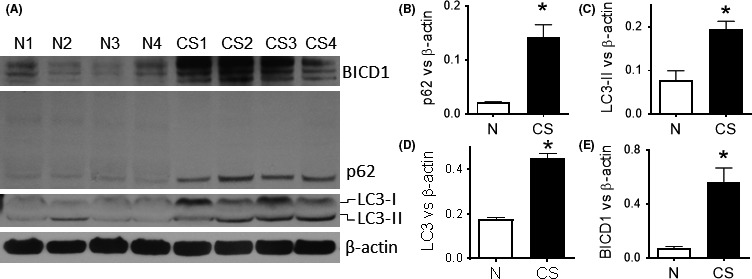
Defective autophagosome maturation in a chronic mice‐smoking model. A/J mice were exposed to cigarette smoke (or fresh air) for 30 min/d for 12 days using Hi‐lite cigarettes. Lungs were removed and homogenized for immunoblotting. Western blot was performed and membranes were immune‐stained against p62 (B), LC3(C,D), BICD1 (E), and β‐actin. N1‐N4: Control mice, CS1‐CS4: smoking mice. Protein amounts were calculated against β‐actin and plotted in graphs. Data were analyzed by using *t* test. *P* < .05 was considered statistically significant. * *P* < .05, n = 4

As shown in Figure 7A,B, p62 monomers accumulated in the lungs of mice that were exposed chronically to CS although no evidence of p62 oligomers was visible. CS also induced LC3‐II suggesting that autophagosomes are present in the lung tissue (Figure 7A,C). Overall LC3 expression was also significantly increased, as observed in COPD, thus suggesting a possible block in autophagy maturation (Figure 7A,D). In agreement with the data observed in COPD biopsies and HC‐CSE experiments, the protein levels of BICD1 were significantly increased in all CS‐exposed mice (Figure 7A,E).

### 3.8 Cardiac glycosides reverse defective autophagosome maturation

**Figure 8:**
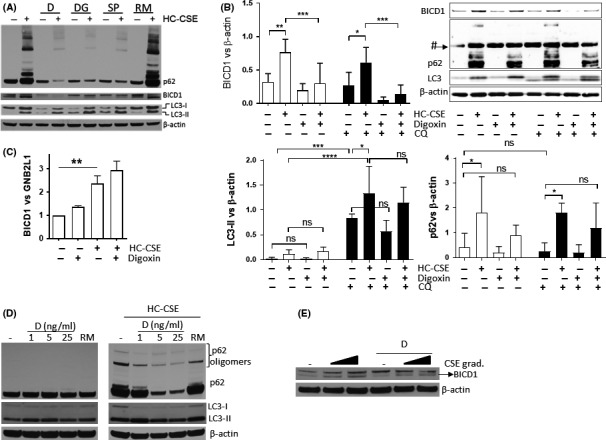
Cardiac glycosides reverse defective CSE‐induced defective autophagosome maturation. (A) BEAS‐2B cells were preincubated either with digoxin (D), digoxigenin (DG), and strophanthidin (SP) or with rapamycin for 6 hours before treatment with HC‐CSE for 24 h. Western blot was performed and membranes were immune‐stained against p62, BICD1, LC3, and β‐actin. (B) BEAS‐2B cells were preincubated with digoxin (D) for 2 h before a 4 h treatment with CQ. Cells were then treated with HC‐CSE for 24 h. Western blot was performed and membranes were immune‐stained against p62, BICD1, LC3, and β‐actin (# p62 unspecific band detected). N = 3, *P < .05, **P < .01, ***P < .001, **** *P* < .0001. (C) BEAS‐2B cells were preincubated with digoxin (D) and then treated with HC‐CSE (10%) for 24 h. mRNA expression of BICD1 was determined and normalized to GNB2L1 mRNA levels. N = 3, **P < .01. (D) HBECs were preincubated with digoxin (D) at 1, 5, and 25 ng/mL prior to treatment with LC‐ (20%) and HC‐ (30%) CSE for 24 h. Western blot was performed and membranes were immune‐stained against p62, LC3, and β‐actin. (E) HBECs were preincubated with 25ng/mL of digoxin (D) prior to treatment with LC‐ (20%) and HC‐ (30%) CSE for 24 h. Western blot was performed and membranes were immune‐stained against BICD1 and β‐actin

In order to reverse defective autophagic flux caused by ineffective autophagy maturation, the effect of cardiac glycosides, previously identified as possible inducers of autophagosome maturation,^37^, ^38^ was investigated and compared to rapamycin. BEAS‐2B cells, preincubated with the cardiac glycosides strophanthidin (SP), digoxin (D), and digoxigenin (DG) (all at 100 ng/mL) prior to HC‐CSE, prevented p62 oligomer formation and an increase in p62, and also reduced HC‐CSE‐induced BICD1 (Figure 8A). In contrast, rapamycin did not prevent induction of p62 and p62 oligomers (Figure 8A). In addition, rapamycin (20 µmol/L), which increased LC3‐II, did not reduce BICD1 levels and even further increased p62 expression (Figure 8A). The effect of cardiac glycosides on autophagic induction by LC3‐II formation was not clear and further investigated using an LC3‐turnover assay. The “LC3‐turnover” assay was performed using CQ in order to see the effects of a digoxin on the autophagic flux. We showed that under defective autophagic flux (CQ treatment) digoxin did not further increased LC3‐II with or without HC‐CSE suggesting that autophagy initiation is not activated by cardiac glycosides (Figure 8B). We also confirmed that despite LC3‐II accumulation by HC‐CSE, this is further increased after CQ treatment suggesting that initiation of autophagy is not affected by HC‐CSE as shown in Figure 2 (Figure 8B). Interestingly, HC‐CSE‐induced p62 (*P* < .05) is not significantly upregulated with digoxin preincubation suggesting that autophagosome maturation restoration must be involved as autophagosomes are not induced by digoxin (Figure 8B). Whereas CQ treatment shows a significant increase in autophagosomes (LC3‐II), it is not the case for p62 suggesting that baseline autophagy in BEAS2B cells turns over p62 slowly (Figure 8B). We confirmed that digoxin significantly reduced HC‐CSE‐induced BICD1 implying that cardiac glycosides restore autophagosome maturation by decreasing BICD1 expression (Figure 8B). Interestingly, despite an increase in BICD1 mRNA transcripts by HC‐CSE, digoxin does not affect BICD1 transcription levels (Figure 8C).

We confirmed our findings in HBECs where preincubation with increasing concentrations of digoxin (1‐25 ng/mL), but not with rapamycin, prevented HC‐CSE‐induced p62 monomer and oligomer accumulation in addition to partly restoring baseline levels of CSE‐induced LC3 (Figure 8D). CSE also induced a concentration‐dependent increase in BICD1 (lower band) that was reduced by preincubation with digoxin (Figure 8E).

An increase in total LC3 and LC3‐II has been associated with autophagic‐induced apoptosis and emphysema via caspase‐3 activation.^6^ We showed that cardiac glycosides completely inhibited HC‐CSE‐induced cleaved caspase‐3 whereas rapamycin only partially prevented this (Figure S9). Interestingly, SMER28, a compound known to induce the clearance of mutant huntingtin fragments independent of mTOR inhibition, also cleared p62 oligomers (Figure S10).^39^ SMER28 also prevented HC‐CSE‐induced cleaved caspase‐3 as did strophanthidin but not rapamycin (Figure S10). Also, SMER28 treatment did not reduce BICD1 protein supporting the concept of a different mechanism of action (Figure S11).

## 4 DISCUSSION

Increasingly, defects in autophagosome maturation are being associated with various pathologies, particularly in neurodegenerative disorders such as Alzheimer's disease, Hungtington's chorea, and Parkinson's disease.^40^ In addition, defective autophagosome maturation has been associated with the aging of the human vascular system, as demonstrated by accumulation of autophagosomes (LC3‐II) with increased total LC3 levels as well as p62.^41^ Recently, aggresome bodies have been described in the lung of COPD patients, together with LC3‐II and p62.^2^, ^3^ We have confirmed that patients suffering from COPD show elevated levels of p62, LC3, and LC3‐II, suggesting that increased presence of autophagosomes is not indicative of high level activation of autophagy but on the contrary, the result of defective autophagosome maturation. Our in vitro data using bronchial epithelial cells that were treated with the main causative factor of COPD, cigarette smoke support this hypothesis. CSE activated the autophagy via an oxidative stress mechanism possibly involving cysteine protease Atg4 inhibition allowing LC3‐I to be converted into LC3‐II.^42^ An increase in LC3‐II with low concentration of CSE was not followed by a decreased p62 protein levels possibly due to the fact that oxidative stress induces p62 transcription levels as shown recently.^43^ Only at higher concentration of CSE did we see a partial reduction of p62 protein 5 hours after incubation. This might represent a state where the degradation of p62 by autophagy was higher than the rate of p62 transcription. High concentrations of CSE induced accumulation of total LC3, p62, and p62 oligomers even after cells were washed and incubated in fresh media. This was associated with persistent presence of autophagosomes as observed in diseases associated with defective autophagosome maturation and in COPD. On the contrary, a lower concentration of CSE only induced transient activation of LC3‐II and p62 oligomers that returned to baseline levels over time. Autophagosome maturation includes the binding between autophagosomes and lysosomes to form autophagolysosomes. We confirmed these findings using HEK293 cells that overexpress the tandem GFP‐mCherry‐LC3 where lower concentrations of CSE induced transient autophagosomes that became autophagolysosomes over time. This was not the case with higher concentrations of CSE where increased numbers of autophagosomes failed to become autophagolysosomes, suggesting a mechanism where lysosomes fail to bind autophagosomes. The different effects of differing concentrations of CSE on the autophagy machinery is cell‐dependent and might be dependent on how much the machinery can cope with oxidative stress‐induced oxidized proteins and organelles which need to be degraded to maintain cellular homeostasis.

We have shown for the first time that BICD1 protein is significantly increased in both mild and severe COPD as compared to non‐COPD smokers, suggesting this increase is an early event in COPD. Also, increased BICD1 was associated with increased lung obstruction and markers of defective autophagosome maturation (LC3, p62, and p62 dimers). In vitro data using BEAS‐2Bs confirmed that only at a high concentration of CSE, which induces defective autophagosome maturation, BICD1 protein was increased, alongside persistent LC3‐II and p62 expressions. It was also observed that CSE induced increased BICD1 transcript levels, and inhibition of transcription by actinomycin D prevented CSE‐induced BICD1 mRNA expression, indicating increased transcription initiation by CSE as opposed to interference with RNA degradation mechanisms. These findings were supported by data obtained in primary bronchial epithelial cells. Mice chronically exposed to CS also displayed increased lung expression of BICD1, which also correlated with accumulation of p62 and total LC3. Knockdown of BICD1 confirmed that CSE‐induced BICD1 is most likely responsible for an accumulation of p62 due to defective autophagosome maturation. Overexpression of the C‐terminal domain of BICD1, which can interact with Rab6A (a protein involved in regulation of protein transport), but not with cytoplasmic dynein, has been shown to alter microtubule movement of Rab6A vesicles inducing an accumulation of these vesicles in the cell.^20^ Recent studies have suggested that BICD1 is unlikely to be involved in lysosome biogenesis or function, but plays an important role in the regulation of endosome trafficking of cargoes to the lysosome.^22^ An accumulation of BICD1 may alter normal trafficking of the autophagosome to the lysosome preventing fusion and resulting in impaired autophagic flux. Our clinical data suggest that accumulation of BICD1 is an early event in COPD and our in vitro data suggest that high concentrations of CSE can cause this accumulation. The reason for the lower levels of BICD1 observed in non‐COPD smokers is not related to the smoking history that is similar between these groups. All non‐COPD except one were current smokers before lung samples were collected. It is possible that some individuals have a propensity to induce BICD1, which could deregulate the normal flux of autophagy thus accumulating cellular damage over time.

Inhibition of autophagy typically results in the accumulation of p62, leading to the formation of large aggregates positive for p62 and ubiquitinated proteins.^44^ CSE‐induced p62 oligomer formation is possibly linked with aggresome formation previously observed in lung from COPD patients.^2^ These oligomers might sequester autophagosomes and autophagic cargo into structures physically too large to degrade, thus bloating the autophagy machinery, disrupting homeostasis, and compromising viability. Formation of SDS‐resistant p62 oligomers has previously been observed with arsenic exposure and in macrophages from smokers.^33^, ^45^ Both studies showed that p62 oligomers were associated with ubiquitinated substrates. More recently, Donohue et al^34^ showed that the autophagy inhibitor verteporfin induces p62 dimers via a mechanism involving low‐level singlet oxygen production. In fact hydrogen peroxide mimicked p62 dimer formation but without p62 monomer accumulation.^34^ We confirmed that CSE induced p62 oligomers in a concentration‐ and time‐dependent manner. At low concentrations of CSE, p62 oligomers were transient, reflecting a functional increased autophagic flux. At higher CSE, p62 oligomers were still present in the cells despite washing‐out CSE. Immunoprecipitation showed that p62 was heavily associated with ubiquitinated substrates after CSE treatment and that deubiquitination by USP2 did not affect oligomer formation. p62 was found to be associated with LC3‐II, as expected, but also with cytoplasmic LC3‐I. This is further proof that oligomers and LC3‐I might exist as aggresomes inside cells. CSE did not further enhance p62 association with the adaptor protein NBR1 and neither did it induce NBR1 SDS‐PAGE resistant oligomers. It is possible that p62 oligomers can affect the flux of autophagy. Donohue et al^34^ showed that oxidant‐induced p62 oligomers needed a functional PB1 domain, thus suggesting that previous aggregation was necessary. We did not confirm these findings and identified the amino acid region of p62 most likely to be involved in oligomer formation as the KIR. Knockdown of Keap1 clearly decreased CSE‐induced oligomer formation and in A549 cells, which have mutant Keap1 that is unable to bind to p62, there were no oligomers after CSE exposure. Mutation of the KIR domain also reduced CSE‐induced oligomers. In fact, NBR1, which lacks a KIR domain, also failed to form oligomers. The exact reason as to why the interaction of p62 with Keap1, which is based on hydrogen bonds, results in these oligomers is not yet understood.

Further proof that CSE induced defective autophagosome maturation was provided by cardiac glycosides. Two studies identified cardiac glycosides as potential compounds that induce autophagosome maturation.^37^, ^38^ In addition, digoxin was also found to be able to induce autophagosomes via activation of the AMPK pathway resulting in mTOR inhibition.^46^ We could not confirm that cardiac glycosides induce LC3‐II formation using an “LC3 turnover assay”; however, we showed how they reduced HC‐CSE‐induced p62 and p62 oligomers suggesting that they target autophagosome maturation. In contrast, an mTOR inhibitor, rapamycin, known to induce autophagosome formation, was unable to clear p62 oligomers via autophagy. We also showed how cardiac glycosides reduced BICD1 expression suggesting a mechanism by which autophagosome maturation is restored under HC‐CSE. Cardiac glycosides increase the level of calcium by inhibiting the Na^+^/K^+^‐ATPase, resulting in augmented contractile force in cardiomyocytes.^47^ In fact, intracellular calcium induces autophagy.^48^ The use of cardiac glycosides has been mainly suggested for cancer therapy, and their stimulatory effect on autophagy may be important in this context.^49^ Recently, cardiac glycosides have been shown to inhibit Tat‐Beclin1‐induced autophagic‐mediated cell death, known as autosis.^50^ However, digoxin had no effect on staurosporine‐induced apoptotic death.^50^ We found that CSE‐induced cleaved caspase‐3 was reversed by cardiac glycosides, including digoxin, whereas rapamycin had no effect. Another cardiac glycoside, bufalin, can inhibit tumor necrosis factor (TNF), IFN, and CXCL‐8.^51^ These novel anti‐inflammatory effects could be related to the modulation of autophagic flux and thus relevant to COPD that is associated with chronic inflammation and autoimmunity.^12^

**Figure 9:**
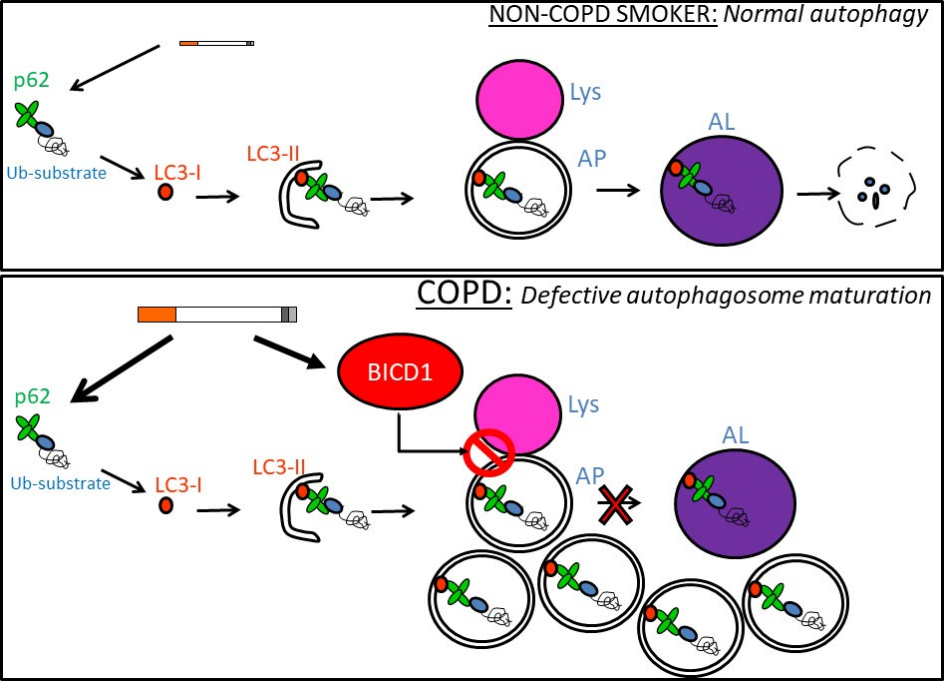
Defective autophagosome maturation hypothesis in chronic obstructive pulmonary disease (COPD). In non‐COPD smokers, CSE induces ubiquitinated proteins that are recognized by p62 and driven toward LC3‐II‐containing double membranes. These double membranes close to become autophagosomes (APs) thus engulfing p62 with ubiquitinated proteins. APs then bind lysosomes (lys) in order to become autophagolysosomes (ALs) and degrade intracellular cargo via lysosomal hydrolases. In COPD, BICD1 accumulates due to excessive CSE exposure over a lifetime. Baseline or CSE‐induced autophagosomes accumulate due to BICD1 preventing autophagosome to lysosome fusion

We have identified BICD1 as novel candidate for defective autophagosome maturation and shown that in COPD, increased BICD1 levels could explain elevated levels of autophagosomes and p62 (Figure 9). Previous research in COPD has also identified the presence of LC3‐II in lung tissue suggestive of an activation of autophagosomes.^6^, ^8^ However, these studies did not measure overall LC3, which also appeared to be increased, as well as p62 levels. The latter is known to negatively correlate with autophagy activation. Increased p62 monomer and oligomer accumulation causes protein aggregates that in turn could result in toxicity for cells as homeostasis is compromised. In fact, protein aggregation, which has been observed in COPD patients, could be caused by defective autophagosome maturation as also observed in several neurodegenerative diseases.^2^, ^7^, ^40^ We have also identified cardiac glycosides as compounds able to restore autophagosome maturation. Thus, cardiac glycosides are possible target for novel drugs that can reverse defective autophagic flux and offer a potential future therapeutic approach in COPD. Further studies would be needed to establish which cell types in the lung of COPD patients are responsible for increased BICD1 expression and defective autophagosome maturation and follow‐up studies in GFP‐LC3 transgenic mice would be required to verify if cardiac glycosides do restore normal levels of autophagy in a COPD model.

## Conflict of Interest

No conflict of interest for any author.

## AUTHOR CONTRIBUTIONS

NM, TJ, KI, ST, and PJB designed the research. NM, TC, CC, CV, YC, and JRB performed the research. TJ and PJB contributed the reagents. NM and TC analyzed the data. NM, TC, PJB, and TJ wrote the manuscript.
