# Supplementary material for: Bicaudal D1 impairs autophagosome maturation in chronic obstructive pulmonary disease
Source: FASEB Bioadv. 2019 Oct 29;1(11):688–705. doi: 10.1096/fba.2018-00055 (PMC6996363; doi:10.1096/fba.2018-00055)

Supplementary Figure Legends

Fig. S1. Effect of current cigarette smoking (n=10) versus ex/non smokers (n=32) on protein markers of autophagy (LC3-II, BICD1, total LC3, p62).

Fig. S2. Effect of CSE on cell viability using MTT. BEAS-2B cells were treated with increasing concentration of CSE (3.3 to 10% v/v) for 24h and an MTT was performed. * $p<0.05$, *** $p<0.0001$, n=3.

Fig. S3. Effect of CSE on caspase-3 activation. Whole-cell extracts from BEAS-2B cells treated with increasing concentration of CSE (3.3 to 10% v/v) for 24h were analysed by immunoblotting for caspase-3, cleaved caspase-3 and β-actin.

Fig. S4. Effect of N-acetylcysteine (NAC) on CSE induced p62. BEAS-2B cells were pre-incubated with NAC (10mM) for 30 min prior to stimulation with LC- and HC-CSE (3.3% and 10%) for 24h. Western blot was performed and membranes were immune-stained against LC3, p62 and β-actin.

Fig. S5. LC3-turnover assay using bafilomycin. An “LC3 turnover” assay was performed by incubating BEAS-2B cells with HC-CSE for 24h plus 2h with bafilomycin (Baf, 200nM). The amount of LC3-II was plotted against β-actin.

Fig. S6. BEAS-2B cells were incubated with HC-CSE (10%) for 24h and p62 was immunoprecipitated from whole cell extracts in the presence of NEM using magnetic Protein A beads. Immunoprecipitated p62 species were resolved by SDS-PAGE followed by Western blot and membranes immune-stained against NBR1 and p62. IgG control (no whole-cell lysates used).

Fig. S7. Effect of CSE on p62 in A549 cells. Whole-cell extracts from A549 cells were treated with increasing concentrations of CSE (10 to 60% v/v) for 24h and analysed by immunoblotting for LC3, p62 and β-actin.

Fig. S8. BEAS-2B cells were pre-incubated with actinomycin-D (5µM) for 10min prior to stimulation with LC- and HC-CSE (3.3 and 10%) for 24h. Western blot was performed and membranes were immune-stained against LC3, BICD1 and β-actin.

Fig. S9. BEAS-2B cells were pre-incubated either with 100ng/ml of digoxin (D), digoxigenin (DG) and strophanthidin (SP) or with 20µM of rapamycin for 6h before treatment with HC-CSE (10%) for 24h. Western blot was performed and membranes were immune-stained against caspase-3, cleaved caspase-3 and β-actin.

Fig. S10. Effect of Strophanthidin (SP), rapamycin (RM) and SMER28 on markers of autophagosome maturation. BEAS-2B cells were pre-incubated either with 100ng/ml of strophanthidin (SP), 20µM of rapamycin or 50µM of SMER28 for 6h before treatment with HC-CSE for 24h. Western blot was performed and membranes were immune-stained against p62, caspase-3, cleaved caspase-3, LC3 and β-actin.

Fig. S11. Effect of SMER28 on markers of autophagosome maturation. BEAS-2B cells were pre-incubated either with 50µM of SMER28 for 6h before treatment with HC-CSE for 24h. Western blot was performed and membranes were immune-stained against BICD1 and β-actin.

S1

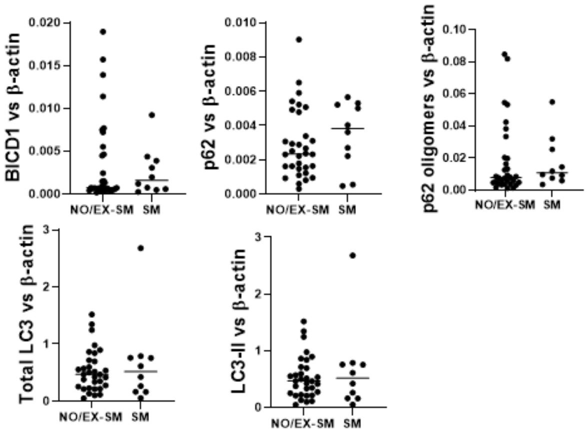

S2

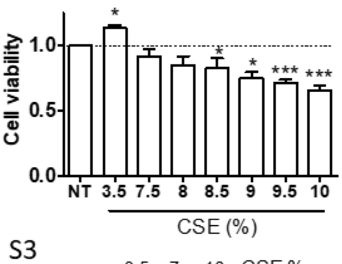

S3

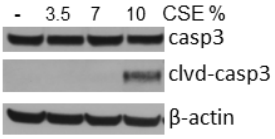

S4

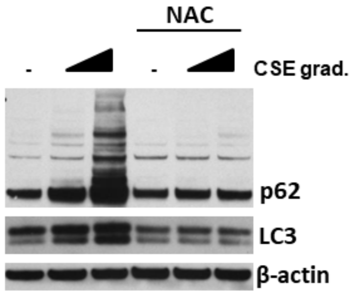

S5

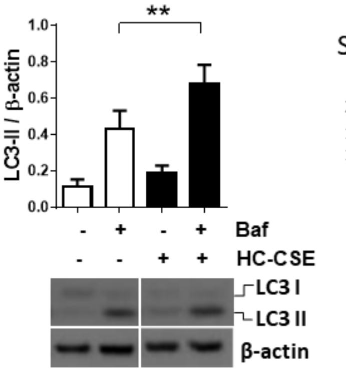

S6

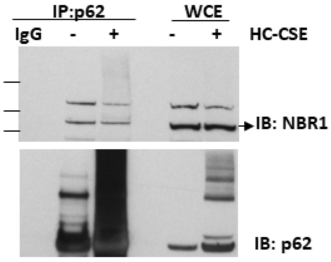

S7

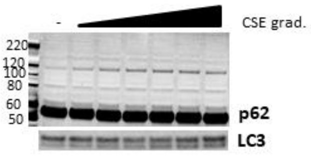

S8

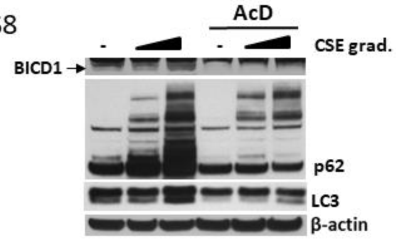

S9

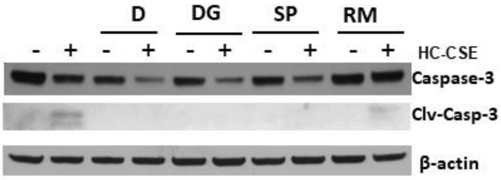

S10

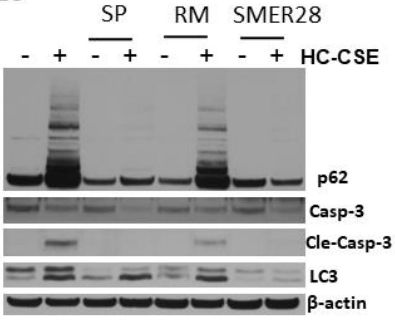

S11

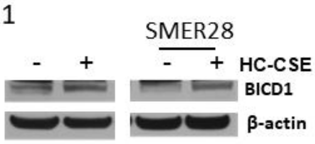

Supplement: Supplementary file 1 [file FBA2-1-688-s001.pdf]
